# Management and glycemic control of patients with type 2 diabetes mellitus at primary care level in Kedah, Malaysia: A statewide evaluation

**DOI:** 10.1371/journal.pone.0223383

**Published:** 2019-10-03

**Authors:** Sharifah Saffinas Syed Soffian, Shahrul Bariyah Ahmad, Huan-Keat Chan, Shahrul Aiman Soelar, Muhammad Radzi Abu Hassan, Norhizan Ismail

**Affiliations:** 1 State Health Department, Ministry of Health Malaysia, Alor Setar, Kedah, Malaysia; 2 Clinical Research Center, Hospital Sultanah Bahiyah, Ministry of Health Malaysia, Alor Setar, Kedah, Malaysia; Mexican Social Security Institute, MEXICO

## Abstract

**Introduction:**

While Kedah has recorded the highest prevalence of diabetes among all the states in Malaysia, the information on the practice and effectiveness of disease management in public health institutions remains limited. This study aimed to evaluate the management and glycemic control of patients with type-2 diabetes mellitus (T2DM) at the primary care level in Kedah.

**Methods:**

All T2DM patients, who made at least one visit to any of the 58 public health clinics in Kedah during August 2016 and July 2017, were included in this study. The sample was selected from the National Diabetes Registry using the stratified random sampling method. The information on the demographic and clinical characteristics, laboratory findings and pharmacological treatment was gathered from medical records of patients. The differences in mean HbA1C levels across subgroups of each variable were tested using the general linear model. The evaluation of the appropriateness of treatment was performed based on the recommendations of the latest Clinical Practice Guidelines for T2DM.

**Results:**

The patients (n = 23,557) were mainly female (63.4%), of Malay ethnicity (80.1%) and middle-aged (62.2%), with a mean duration of T2DM of 6.2±7.16 years. Only 15.6% of them had a HbA1C level <6.5%, and 28.6% did not have their HbA1C levels tested over the 12-month period. Yet, the underutilization of combination treatment (≥2 antidiabetic agents) and insulin in the patients with a poor glycemic control was evident. Retinopathy emerged as the most prevalent diabetes-related complication (12.6%). Along with those with a longer duration of T2DM, the patients who were younger, female and of Indian ethnicity were found to generally have a poorer glycemic control.

**Conclusion:**

This study discloses the suboptimal T2DM management at the primary care level in Kedah, which warrants a statewide plan for improvement.

## Introduction

Diabetes is currently a public health concern worldwide. Over the last 25 years, the global prevalence of diabetes increased from 4.3 to 9% in men, and from 5 to 7.9% in women [1]. Consistent with the global trend, the prevalence of type 2 diabetes mellitus (T2DM) in Malaysia also grew from 11.6 to 17.5% in the last decade. The number of T2DM patients in the country is projected to reach 2.48 million by 2030 [2,3]. As one of the major non-communicable diseases, diabetes has been posing a major challenge to the Malaysian public health system, incurring an additional healthcare cost of USD 1.07 to 1.83 (MYR 4.49 to 7.67) million annually [4].

A wide range of complications of poorly controlled T2DM have been claiming nearly 5 million lives each year [5]. The common microvascular complications include nephropathy, retinopathy and neuropathy, while the common macrovascular complications include stroke, cardiovascular disease and peripheral artery disease [6]. Other unclassified complications, such as diabetic foot syndrome and reduced resistance to infections, have also significantly affected the quality of life of T2DM patients and resulted in deaths [7,8].

Within this context, a tight glycemic control with a glycosylated hemoglobin (HbA1C) level ≤7% is recommended for T2DM patients in general, while a more stringent glycemic control with a HbA1C level ≤6.5% is desirable for those who are younger, newly diagnosed and without complications [9]. Yet, maintaining the glycemic control, optimizing the treatment, and ensuring the patient adherence consistently prove to be the major challenges in T2DM management globally [10–12]. In a US large-scale cross-sectional study, only 31.4% of patients who had any insulin use were shown to have a HbA1C level ≤7% [13]. However, although most T2DM patients have a suboptimal glycemic control, health practitioners are likely not to have a plan to intensify their treatment [14]. Furthermore, nonadherence to treatment in 7 to 64% of T2DM patients has been reported [15].

Kedah, a northern state with a population of nearly two million, has recorded the highest prevalence of diabetes in Malaysia. Approximately one in every four residents in this state are living with diabetes [16]. To date, public health clinics remain the main providers of primary health care in Malaysia [17]. However, the information on how T2DM has been managed in these settings against the backdrop of scarce resources is still limited. As more aggressive treatment with the early use of combination treatment and insulin is recommended by the latest Clinical Practice Guidelines (CPG) for T2DM [18], this study was designed to examine the current management and glycemic control of T2DM patients seeking care at public health clinics across Kedah.

## Materials and methods

### Study design and ethics approval

This was a cross-sectional study. The data was contributed by 58 public health clinics located in Kedah. The permission to collate the data from the clinics was sought from the Public Health Division of the State Health Department of Kedah, while the study proposal (NMRR-17-2040-37634) was reviewed and approved by the Medical Research Ethics Committee, Malaysia.

### Study population and sampling

The study population consisted of patients who i) were diagnosed with T2DM, and ii) made at least one visit to any of the public health clinics in Kedah between 1^st^ August 2016 and 31^th^ July 2017. The minimal sample size required was 18,774, calculated based on the estimated total number of T2DM patients in Kedah (400,000 or 25% of an adult population of 1.6 million) with the confidence level and margin of error fixed at 99.5 and 1%, respectively [16,19]. The targeted sample size was increased to 22,529 to compensate for incomplete data in an estimated 20% of the patients. The sampling frame was obtained from the web-based National Diabetes Registry (NDR) [20], which was shown to have captured 90.2% of the T2DM patients receiving treatment at the public health clinics in a statewide internal audit in 2017. The sample was acquired using the stratified random sampling method according to the proportion of eligible patients contributed by each of the 58 clinics, and the list of randomly selected patients for each clinic was autogenerated by the NDR.

### Data collection and assessment

A standardized case report form was developed for data collection from the medical records of the selected patients. The information on age, gender, ethnicity, body mass index (BMI), waist circumference, duration of T2DM, comorbidities (hypertension and dyslipidemia), the latest HbA1C level (tested during 1^st^ August 2016 to 31^th^ July 2017), pharmacological treatment, and the presence of diabetes-related complications (retinopathy, nephropathy, ischemic heart disease, cerebrovascular disease and diabetic foot ulcer) was gathered.

The HbA1C levels of patients were further categorized into five groups according to the cut-offs set by the CPG (<6.5, 6.5–7.4, 7.5–8.4, 8.5–10.0, and >10.0%), which were recommended to be used in guiding the treatment selection. The appropriateness of the pharmacological treatment was also evaluated based on the recommendations of the CPG: i) lifestyle approach or monotherapy (without insulin) for a HbA1C level <6.5%, (ii) monotherapy (without insulin) for 6.5–7.4%, (iii) dual combination therapy (with or without insulin) for 7.5–8.4%, (iv) triple combination therapy (with or without insulin) for 8.5–10.0%, and (v) insulin-based combination therapy for >10% [18].

### Statistical analysis

The data analysis was performed using the R-3.5.1 for Windows. All the categorical variables were summarized as frequencies and percentages, and numerical variables as means and standard deviations (SDs). The differences in mean HbA1C levels across subgroups of each variable were tested using the general linear model adjusted for all the remaining variables. The significant levels of all the statistical tests were fixed at 5%.

## Results

Over the stipulated 12-month period, a total of 80,028 eligible patients were identified. Of the 23,557 patients included in this study, the majority were female (63.4%) and Malay (80.1%). Approximately 60% of them were in the age range of 40 to 60 years, and 11.3% were aged under 40 years. More than half of them were found to be overweight (BMI 25.0–29.9kg/m^2^) or obese (BMI >30.0kg/m^2^), and only 25.8% had a normal BMI. On average, they had been diagnosed with T2DM for 6.2±7.16 years. Nearly 71.4% of them had their HbA1C levels tested during the 12-month period, with a mean HbA1C level of 8.4±2.23%. Meantime, only 15.6% of them were found to have an optimal glycemic control with a HbA1C level <6.5%. Most of them were also reported to have hypertension (75.8%) and dyslipidemia (74.1%). Retinopathy (12.6%) emerged as the most common diabetes-related complication, followed by nephropathy (10.1%) and ischemic heart disease (6.6%). Approximately 90% of the patients received pharmacological treatment, and more than half received combination treatment (≥2 antidiabetic agents). Nevertheless, insulin was only used in 26.4% of them (Table 1).

**Table 1:** Demographics, clinical profile, glycemic control and treatment of patients (n = 23,557).

Variables		n	(%)	Mean	(SD)
					
**Age, years**					
	<30	436	(1.9)		
	30–39	2,210	(9.4)		
	40–49	6,220	(26.4)		
	50–59	8,436	(35.8)		
	60–69	4,760	(20.2)		
	≥70	1,495	(6.3)		
**Gender**					
	Male	8,626	(36.6)		
	Female	14,931	(63.4)		
**Ethnicity**					
	Malay	18,864	(80.1)		
	Chinese	2,130	(9.0)		
	Indian	2,144	(9.1)		
	Others	419	(1.8)		
**BMI, kg/m^2^**					
	<18.5	390	(1.7)		
	18.5–24.9	6,070	(25.8)		
	25.0–29.9	7,557	(32.1)		
	30.0–34.9	3,789	(16.1)		
	35.0–39.9	1,226	(5.2)		
	≥40.0	394	(1.7)		
	Undocumented	4,131	(17.5)		
**Duration of T2DM, years**				6.2	(7.16)
**HbA1C, %**				8.4	(2.23)
**HbA1C level, %**					
	<6.5	3,674	(15.6)		
	6.5–7.4	3,580	(15.2)		
	7.5–8.4	2,640	(11.2)		
	8.5–10.0	3,191	(13.5)		
	>10.0	3,725	(15.8)		
	Not tested during study period	6,747	(28.6)		
**Hypertension**		17,854	(75.8)		
**Dyslipidemia**		17,450	(74.1)		
**Diabetes-related complications**					
	Retinopathy	2,968	(12.6)		
	Nephropathy	2,379	(10.1)		
	Ischemic heart disease	1,554	(6.6)		
	Cerebrovascular disease	447	(1.9)		
	Diabetic foot ulcer	259	(1.1)		
**Treatment**					
	Lifestyle approach	2,503	(10.6)		
	Metformin only	4,964	(21.1)		
	Sulfonylurea only	982	(4.2)		
	Any other single OAD	193	(0.8)		
	Two or more OADs	8,700	(36.9)		
	Insulin only	1,496	(6.4)		
	Insulin and OADs	4,719	(20.0)		

BMI, body mass index; SD, standard deviation; T2DM, type 2 diabetes mellitus; HbA1C, glycosylated hemoglobin; OAD, oral antidiabetic drug.

In the patients whose HbA1C levels were tested at least once during the 12-month period (n = 16,810), the glycemic control was shown to improve with age. Nearly half of the patients under 30 years of age had a HbA1C >10.0%, while more than 40% of the patients above 70 years of age had a HbA1C level <6.5%. All the age groups also had a significantly lower mean HbA1C level than did the under-30 group, except for the 30–39 group. Men were shown to have a slightly lower mean HbA1C level than did women (8.1% vs. 8.3%; p<0.001). It is also noted that the mean HbA1C level of the Malay patients was lower than that of the Indian patients (8.4% vs. 8.6%; p<0.001) but higher than that of the Chinese patients (8.4% vs. 7.8%; p<0.001). The targeted HbA1C level of 6.5% was achieved in approximately 30% of the Chinese patients but only in 14.8% of the Indian patients. BMI was not found to be associated with the HbA1C level. However, the glycemic control was found to deteriorate with time, indicated by a slightly higher mean HbA1C level in those who had been having T2DM for more than 6.2 years (8.2% vs. 8.1%; p = 0.034). The patients who had hypertension were also found to have a slightly better glycemic control level than did those who did not have hypertension (8.1% vs. 8.3%; p<0.001). However, the patients with dyslipidemia demonstrated an opposite trend, showing a slightly higher mean HbA1C level as compared with those without dyslipidemia (8.3% vs. 8.1%; p<0.001) (Table 2).

**Table 2:** Distribution and adjusted means of HbA1C Levels across patient subgroups (n = 16,810).

Variables		HbA1C level (%)												p-value
		<6.5		6.5–7.4		7.5–8.4		8.5–10.0		>10.0		β (95% CI)	Adjusted mean (SE)^a^	
		n	(%)	n	(%)	n	(%)	n	(%)	n	(%)			
**Age groups, years**														
	<30	38	(12.9)	37	(12.5)	35	(11.9)	50	(16.9)	135	(45.8)	Reference	8.7 (0.13)	
	30–39	165	(10.8)	200	(13.1)	203	(13.3)	369	(24.1)	592	(38.7)	0.00 (-0.26, 0.25)	8.7 (0.07)	0.971
	40- 49years	657	(14.5)	756	(16.7)	712	(15.8)	1,041	(23.0)	1,353	(29.9)	-0.34 (-0.58, 0.10)	8.4 (0.06)	0.005
	50- 59years	1,308	(21.3)	1,388	(22.6)	1,038	(16.9)	1,184	(19.3)	1,223	(19.9)	-0.70 (-0.94, 0.46)	8.0 (0.06)	<0.001
	60-69years	1,102	(32.6)	927	(27.4)	528	(15.6)	472	(14.0)	352	(10.4)	-1.03 (-1.28, 0.78)	7.7 (0.06)	<0.001
	≥70years	404	(42.8)	272	(28.8)	124	(13.1)	75	(7.9)	70	(7.4)	-1.16 (-1.43, 0.89)	7.6 (0.08)	<0.001
**Gender**														
	Male	1,376	(23.2)	1,281	(21.6)	969	(16.4)	1,146	(19.3)	1,153	(19.5)	Reference	8.1 (0.06)	
	Female	2,298	(21.1)	2,299	(21.1)	1,671	(15.3)	2,045	(18.8)	2,572	(23.6)	0.12 (0.06, 0.18)	8.3 (0.06)	<0.001
**Ethnicity**														
	Malay	2,943	(21.4)	2,843	(20.7)	2,152	(15.7)	2,648	(19.3)	3,161	(23.0)	Reference	8.4 (0.05)	
	Chinese	449	(30.8)	425	(29.1)	222	(15.2)	214	(14.7)	149	(10.2)	-0.52 (-0.62, -0.41)	7.8 (0.07)	<0.001
	Indian	188	(14.8)	237	(18.7)	208	(16.4)	266	(21.0)	367	(29.0)	0.23 (0.12, 0.34)	8.6 (0.07)	<0.001
	Others	94	(27.8)	75	(22.2)	58	(17.2)	63	(18.6)	48	(14.2)	-0.39 (-0.61, -0.17)	8.0 (0.12)	0.001
**BMI ^b^**														
	<18.5	101	(34.4)	56	(19.0)	38	(12.9)	41	(13.9)	58	(19.7)	Reference	8.2 (0.12)	
	18.5–24.9	1,148	(23.9)	1,051	(21.9)	696	(14.5)	860	(17.9)	1,047	(21.8)	0.08 (-0.15, 0.31)	8.3 (0.05)	0.486
	25.0–29.9	1,270	(21.0)	1,258	(20.8)	967	(16.0)	1,167	(19.3)	1,382	(22.9)	-0.01 (-0.24, 0.21)	8.2 (0.05)	0.899
	30.0–34.9	574	(18.7)	667	(21.7)	524	(17.1)	653	(21.2)	655	(21.3)	-0.07 (-0.30, 0.16)	8.2 (0.06)	0.547
	35.0– 39.9	179	(17.8)	232	(23.1)	156	(15.5)	187	(18.6)	250	(24.9)	-0.08 (-0.33, 0.17)	8.1 (0.08)	0.512
	≥40	57	(18.1)	53	(16.8)	66	(21.0)	70	(22.2)	69	(21.9)	-0.14 (-0.45, 0.17)	8.1 (0.12)	0.370
**Duration of T2DM^c^**														
	< 6.2 years	2,328	(26.8)	2,056	(23.7)	1,368	(15.8)	1,456	(16.8)	1,473	(17.0)	Reference	8.1 (0.06)	
	≥ 6.2 years	1,346	(16.7)	1,524	(18.7)	1,272	(15.6)	1,735	(21.3)	2,252	(27.6)	0.07 (0.01, 0.14)	8.2 (0.06)	0.034
**Hypertension**														
	No	658	(16.7)	708	(18.0)	594	(15.1)	776	(19.7)	1,203	(30.5)	Reference	8.3 (0.06)	
	Yes	3,016	(23.1)	2,872	(22.0)	2,046	(15.7)	2,415	(18.5)	2,702	(20.7)	-0.26 (-0.34, -0.18)	8.1 (0.06)	<0.001
**Dyslipidemia**														
	No	924	(22.5)	823	(20.0)	589	(14.3)	749	(18.2)	1,023	(24.9)	Reference	8.1 (0.06)	
	Yes	2,750	(21.2)	2,757	(21.2)	2,051	(15.8)	2,442	(18.8)	2,986	(23.0)	0.19 (0.11, 0.26)	8.3 (0.06)	<0.001
**Diabetic Treatment**														
	Lifestyle approach	301	(54.5)	109	(19.7)	49	(8.9)	50	(9.1)	43	(7.8)	Reference	7.1 (0.10)	
	Metformin only	1,582	(42.7)	1,155	(31.1)	466	(12.6)	274	(7.4)	232	(6.3)	0.05 (-0.14, 0.24)	7.1 (0.06)	0.587
	Sulfonylurea only	275	(37.5)	201	(27.4)	110	(15.0)	100	(13.6)	48	(6.5)	0.37 (0.15, 0.60)	7.4 (0.09)	0.001
	Any other single OAD	23	(15.8)	37	(25.3)	23	(15.8)	27	(18.5)	36	(24.7)	1.27 (0.89, 1.64)	8.3 (0.17)	<0.001
	Two or more OADs	1,201	(17.9)	1,613	(24.0)	1,346	(20.0)	1,446	(21.5)	1,112	(16.6)	1.07 (0.89, 1.25)	8.1 (0.05)	<0.001
	Insulin only	89	(7.9)	137	(12.1)	157	(13.9)	249	(22.0)	499	(44.1)	2.53 (2.32, 2.75)	9.6 (0.08)	<0.001
	Insulin and OADs	203	(5.3)	328	(8.6)	489	(12.8)	1,045	(27.4)	1,755	(45.9)	2.56 (2.37, 2.75)	9.6 (0.06)	<0.001

BMI, body mass index; CI, confidence interval; HbA1C, glycosylated hemoglobin; OAD, oral antidiabetic drug; SE, standard error, T2DM, type 2 diabetes mellitus.

^a^ General linear model adjusted for the remaining variables; performed on 15,532 patients with complete data for all the variables.

^b^ BMI was undocumented for 1,278 patients.

^c^ Dichotomized using the overall mean duration of T2DM as the cutoff.

None of the subgroups divided according to the treatment received was shown to have a mean HbA1C level below 7.0%, let alone the targeted 6.5%. Despite the recommendations of the CPG, it is also found that combination treatment (≥2 antidiabetic agents) was not used in nearly 30% and 25% of the patients with a HbA1C level of 7.5–8.4% and 8.5–10.0%, respectively. Meanwhile, insulin was only given to 60.5% of the patients with a HbA1C level >10.0%, even though its use has been strongly recommended by the CPG (Table 2).

## Discussion

To the best knowledge of the investigators, this is the first large-scale study which examines the sufficiency of the management of T2DM, as well as the glycemic control of patients, at a state level in Malaysia. The strength of this study mainly lies in the use of the stratified random sampling method, which ensured that the patients included were representative of the population receiving treatment at all the 58 public health clinics in Kedah. In addition, the sample was selected from the NDR, which have captured more than 90% of the diabetes patients seeking care from the public health clinics in the state. Therefore, the findings could be used to guide the State Health Department in revising the existing strategies used for diabetes management, particularly at the primary care level.

The results demonstrate that uncontrolled T2DM, indicated by a HbA1C level above 6.5%, occurred in approximately 85% of the patients. This was consistent with the findings of several studies, which suggest that the glycemic control of T2DM patients in Malaysia has been generally suboptimal [2,21,22]. The overall mean HbA1C level found in this study (8.4%) was also slightly higher than those reported based on the NDR for the general population of Malaysia during 2009 to 2012 (8.0 to 8.3%) [20]. While an uncontrolled HbA1C level has been consistently linked to a wide range of complications and increased financial burden, educational and health promotion strategies which were shown to be potentially effective in the local context are warranted [23,24]. Despite the recommendations on regular assessment,^18^ it is also noteworthy that approximately one-third of the patients in this study did not have their HbA1C levels tested over the 12-month period. Hence, it is imperative to improve the access to the routine assessment of HbA1C levels among the public health clinics in Kedah.

The findings also suggest that T2DM patients in Kedah had been undermanaged, even though it is arguable that personalized approach might have been used in diabetes treatment. This is supported by the fact that nearly 40% of the patients with a HbA1C level above 10.0% in the state still did not receive insulin as part of their treatment. Such findings would be expected, as T2DM patients in Malaysia generally have negative perceptions regarding the side effects of insulin, and thus a high tendency of refusing the treatment [25,26]. Aside from that, cost of insulin treatment was highlighted as the major concern of the patients in Malaysia.^26^ Hence, in addition to patient education, there is also a need for a strategy to help reduce the financial burden of T2DM patients, who have been expected to purchase needles for insulin injection, together with glucometers and glucostrips for self-monitoring of blood glucose. Meanwhile, most of the patients who had a HbA1C level above 7.5% did not receive combination treatment. Given that the latest CPG for T2DM has provided clear recommendations for treatment selection based on the glycemic control, its use could be encouraged among the prescribers to optimize the patient management.

Despite the inadequate management of T2DM in general, the prevalence of the corresponding complications is found to be relatively low as compared with the similar studies. For example, the prevalence of retinopathy, one of the most common complications of T2DM, was only 12.6%, while the prevalence reported by similar settings could be as high as 23.8% [27]. This was most likely due to the under-screening of diabetes-related complications. According to the findings of a statewide internal audit in Kedah, more than half of the patients referred for fundus photography-based retinopathy screening or for further examination in a specialist clinic failed to attend the scheduled appointments. While most of the patients were also found to have comorbidities, including hypertension, dyslipidemia and obesity, strategies to scale up the early screening of diabetes-related complications and ensure the proper management of T2DM with evidence-based treatment selection are essential.

Interestingly, women composed approximately two-thirds of the T2DM population in this study, and yet they seemingly had a slightly poorer glycemic control than men. This would be expected, as men have been well known for the delay in seeking health care for their illnesses [28]. Another important issue is the early development of T2DM in the young population. It is found that more than 10% of T2DM patients seeking treatment in Kedah were under 40 years of age. Yet, the glycemic control of the younger patients was shown to be poorer than that of the older patients. Hence, screening programs and interventions planned should also target the younger adults, as the early-onset T2DM is increasingly prevalent globally [29]. Moreover, irrespective of a similar observation reported previously [30], further research is needed to explore the factors which led the patients of Malay and Indian ethnicity to a higher prevalence of T2DM and a poorer glycemic control in general.

Notwithstanding the above findings, this study has several limitations. First, the analysis was limited to the information available in the medical records of patients. Some clinical and non-clinical factors, which could greatly affect the outcomes of T2DM management, such as the adherence to treatment and self-care activities of patients, were not captured in the analysis. The analysis was also limited by incomplete data regarding waist circumference and BMI, which had not been routinely measured in DM patients. Moreover, due to the nature of the cross-sectional design of the study, the impact of the treatment and glycemic control on the development of diabetes-related complications remains unclear.

## Conclusion

The findings of this study indicate that the management of T2DM patients receiving treatment at the public health clinics in Kedah has been suboptimal. Only 15.6% of the patients had a HbA1C level <6.5%, while approximately 30% of them did not have their HbA1C levels tested over the 12-month study period. Yet, a considerable proportion of the patients with a poor glycemic control had received neither combination nor insulin therapy. Specifically, a poorer glycemic control was observed in the younger patients and women. Ethnicity and the duration of T2DM were also shown to be associated with the glycemic control. Overall, the findings necessitate an improvement in the existing practice in diabetes management, particularly at the primary care level.

## Supporting information

S1 Fig Case report form used for data collection. (PDF)
