# Supplementary material for: Management and glycemic control of patients with type 2 diabetes mellitus at primary care level in Kedah, Malaysia: A statewide evaluation
Source: PLoS One. 2019 Oct 3;14(10):e0223383. doi: 10.1371/journal.pone.0223383 (PMC6776298; doi:10.1371/journal.pone.0223383)
Supplement: S1 Fig — (PDF) [file pone.0223383.s001.pdf]

MANAGEMENT AND GLYCEMIC CONTROL OF PATIENTS WITH TYPE 2 DIABETES MELLITUS AT PRIMARY CARE LEVEL IN KEDAH, MALAYSIA: A STATEWIDE EVALUATION

CASE REPORT FORM

A. Demographic characteristics

Patient code: ………………………………………………

Gender: Female / Male

Ethnicity: Malay/ Chinese/ Indian/ Others

Date of birth: ………………………………………………

Date of diagnosis: ……………………………………….

B. Clinical characteristics

Characteristics	Latest Results (1st Aug 2016 – 31st July 2017)	Date	Not performed
Height	cm		
Weight	kg		
Body mass index (BMI)	kg/m^2		
Blood pressure	mmHg		
Random blood sugar	mmol/L		
Fasting blood sugar	mmol/L		
HbA1c	%		
Creatinine	µmol/l		
Microalbuminuria	Positive / Negative		
Proteinuria	Positive / Negative		
Fundus examination	Normal / Abnormal		
Foot examination	Normal / Abnormal		

(c) Diabetes-related complications

Complication	Present	Absent	If PRESENT, date of diagnosis
Retinopathy			
Ischemic Heart Disease			
Cerebrovascular Disease			
Nephropathy			
Diabetic Foot Ulcer			

(d) Concomitant diseases

Diseases	Yes	No	If YES, date of diagnosis
Hypertension			
Dyslipidemia			

(e) Treatment received

Diabetes Medication	Yes
Biguanides (e.g Metformin)	
Sulphonylureas (e.g Glibenclamide)	
Alpha-glucosidase inhibitors (acarbose)	
Meglitinides (e.g repaglinide)	
Glitazones (e.g rosiglitazones)	
Insulin	
Lifestyle approach	
